# Application of Photocatalytic Concrete Paving Blocks in Poland—Verification of Effectiveness of Nitric Oxides Reduction and Novel Test Method

**DOI:** 10.3390/ma13225183

**Published:** 2020-11-17

**Authors:** Hubert Witkowski, Janusz Jarosławski, Anna Tryfon-Bojarska

**Affiliations:** 1 Department of Building Physics and Building Materials, Łódź University of Technology, Al. Politechniki 6, 90–924 Łódź, Poland; 2 Consulting for Construction Hubert Witkowski, 92-511 Łódź, Poland; 3 Institute of Geophysics, Polish Academy of Sciences, Księcia Janusza st. 64, 01–452 Warszawa, Poland; januszj@igf.edu.pl; 4 Innovation Management Unit, SGH Warsaw School of Economics, Al. Niepodległości 162, 02–554 Warszawa, Poland; anna.tryfon@skanska.pl; 5 Skanska Property Poland sp. z o.o., Al. Solidarności 173, 00–877 Warszawa, Poland

**Keywords:** photocatalytic concrete, NO*_x_* reduction, solar UV irradiance

## Abstract

Photocatalytic concrete is one of the most promising concrete technologies of the past decades. Application of nanometric TiO_2_ to cement matrices enables the reduction of harmful airborne pollutants. Although a number of implementations of this technology are described in this paper, problems related to test conditions are also reported. One major issue is the sufficient light irradiation that for higher latitudes can be significantly reduced. In this paper, a field campaign on the implementation of photocatalytic concrete pavement in Warsaw (52.23° N) is briefly described. Based on experience from the field campaign, a novel test method is developed. In the research, the effectiveness of nitric oxide reduction is verified at natural light irradiation for various dates of solar position at noon in central Poland (51.83° N). The results confirm the benefits of using photocatalytic materials at higher latitudinal locations. The experimental setup presented in the study combines the advantages of controlled measurement conditions typical in laboratory tests with the possibility of including natural sunlight conditions in the investigation process.

## 1. Introduction

According to the European Environmental Agency’s 2018 report on air quality in Europe [1], the air pollutants most hazardous to human health are PM’s (particulate matter), NO_2_, and ground-level O_3_. The estimated impact on the population of European countries from exposure to NO_2_ concentration in 2015 alone was approximately 79,000 premature deaths. Consequently, the problem of air quality has become one of major importance.

In urban areas, one of the dominant sources of airborne pollution is traffic. Hence, air quality has been analyzed using direct measurements [2,3,4], as well as numerical simulations [5,6,7]. Analysis of air pollution in Europe described by Koolen and Rothenberg [8] indicated that among a variety of airborne pollutions, reduction of NO*_x_* (a sum of NO and NO_2_) emissions is the most urgent needed response and potentially the most beneficial. The application of photocatalytic materials based on the cement matrix enables the reduction of nitric oxide concentration in the presence of UV light during the process of photocatalysis. Therefore, the number of implementations of this promising technology have increased in the last decade. The wide range of applications includes using vertical elements such as façade panels and coatings as well as horizontal elements such as concrete pavements. Photocatalytic properties in concrete are provided by the addition of a nanometric catalyst to the cement matrix. One widely known and used photocatalyst is nanometric titanium dioxide (TiO_2_). The main advantages of TiO_2_ are: high chemical stability, nontoxicity, and high oxidizing power. TiO_2_ exists in three different forms: anatase, brookite, and rutile. Anatase exhibits the highest overall photocatalytic activity [9]. In the late 1960s, Fujishima and Honda [10] described the mechanism of the photocatalytic reaction of TiO_2_. In the process of photocatalysis, certain atmospheric pollutants can be degraded—such as nitric oxides, volatile organic compounds (VOCs), and non-volatile organic residues—due to charge transfer (redox) on the catalyst surface [11]. The simplified mechanism of nitrogen oxide reduction can be described as follows:

(1) $$\mathrm{NO}+\mathrm{OH}\overset{hV,{\;\mathrm{TiO}}_{2}}{\to }{\mathrm{NO}}_{2}+H^{+}$$

(2) $${\mathrm{NO}}_{2}+\mathrm{OH}\overset{hV,{\;\mathrm{TiO}}_{2}}{\to }{\mathrm{NO}}_{3}^{-}+H^{+}$$

The NO_3_ formed on the photocatalytic concrete surface reacts with the calcium in the cement matrix to form a water-soluble calcium nitrate. Next, the salt is removed with rainwater [12].

The effectiveness of photocatalytic concrete technology has recently been the subject of a number of laboratory and field studies. The most significant factors affecting NO*_x_* degradation are irradiance, relative humidity, pollutant concentration, and flow rate over the photocatalytic surface. In laboratory tests, the impact of various factors has been studied, such as the type and amount of TiO_2_ added to concrete [13], UV radiation wavelength range [14], concrete surfaces [15], humidity [16], temperature [17], and the carbonation process of concrete [18].

Test methods have been described in standards such as: ISO 22197–1 [19], UNI 11247 [20], JIS R 1701–1 [21]. The test setup described in standards is designed to provide a laminar flow of the gas along the surface of sample; therefore, the space between sample’s surface and the glass covering is very narrow (about 3 mm). This system provides the ideal conditions to measure the reduction of NO*_x_* concentration during constant gas flow. However, the real conditions are more complex, which has been confirmed in field studies. A number of projects on the application of photocatalytic materials have been conducted recently across Europe, the United States, and China. Although general observations of field campaigns are in accordance with the findings of laboratory studies, the measured values of pollutant reduction substantially vary in particular cases. The influence of dirt deposits on photocatalytic road pavement in Bergamo resulted in a reduction of approximately 16% photoactivity a few weeks after the completion of the installation of photocatalytic concrete pavement [22]. After one year of service of 10,000 m^2^ photocatalytic pavement blocks on the parking lanes in Antwerp, a 20% reduction in purification efficiency was measured [17]. The application of photocatalytic cement-based paint on walls and ceilings in the “Umberto I” tunnel in Rome resulted in a NO*_x_* reduction of over 20%, measured in two monitoring campaigns [23]. George et al. [24] and Boonen et al. [25] have described no significant pollutant reduction during the field campaign at the Leopold II tunnel in Brussels.

In most cases, in-situ tests have proven the effectiveness of photocatalytic technology in NO*_x_* reduction. However, the results indicated that the ability to reduce pollutants with the use of photocatalytic concrete is highly vulnerable to various external factors such as wind speed, dirt and humidity that occur at the particular moment of testing. Therefore, to achieve reliable data, a long-term campaign is needed.

The first commercial large-scale application of photocatalytic concrete for an office building in the center of Warsaw Generation Park was preceded by research carried out by the Skanska Property Poland and the Górażdże Cement consortium, together with scientific partners: the Institute of Geophysics of Polish Academy of Sciences, the Warsaw University of Technology, and the Geology Department of the University of Warsaw. Although the effectiveness of NO_x_ reduction on the applied concrete blocks was proven in laboratory tests according to standard UNI 11247 [20], the effectiveness of the location of the pavement application had to be verified. The first concern was related to the availability of sufficient solar irradiance to trigger photocatalytic reactions at the latitude of 52.23° N. The next concern was related to land relief around the measurement point—the roundabout is an open crossing of two city traffic arteries vulnerable to wind impact. The target large-scale application was conditioned by the result of the field tests; a minimum 15% of NO*_x_* abatement was an assumed requirement for further implementation of the new technology. For the field campaign, about 350 m^2^ of public sidewalk next to the Daszynskiego roundabout and next to the office building was chosen. In the period from the end of June 2018 to mid-September 2018, several daily tests were conducted. Air quality was measured above both the regular and the photocatalytic pavement. Some selected results of this field campaign are presented in this paper.

Folli and Macphee [26] described a simple model to obtain UV contributions to solar radiation as a function of geography and season. The obtained results indicated that proper UV contribution is achieved throughout the whole year only at latitudes below 35°. The other factor described in the model was the length of a day. Folli and Macphee [26] discussed an example of photocatalytic pavement application in Copenhagen at a latitude of 55.68° N. A significant NO reduction was achieved for a total UV daily dose exceeding 600 kJ/m^2^ day^−1^.

Experience from the field campaign in the Generation Park project and the aspects mentioned above were the motivation to undertake research on a novel test method that would reduce the influence of various external factors (such as wind or variable pollutant concentration) and would reflect a natural UV irradiance occurring in the considered location. In the paper, a novel method of effective air purification and results of NO*_x_* reduction measured at an autumnal period at Central Geophysical Observatory of Institute of Geophysics Polish Academy of Sciences (Belsk, Poland, 51.83° N; 20.78° E) are presented. The results obtained were compared with the laboratory test method described in UNI 11247 [20].

Properties of applied photocatalytic cement were examined with a scanning electron microscope (SEM) using SEM elemental mapping. The main objective of this research was to verify the distribution of nanoparticles in cement.

## 2. Materials

**Figure 1:**
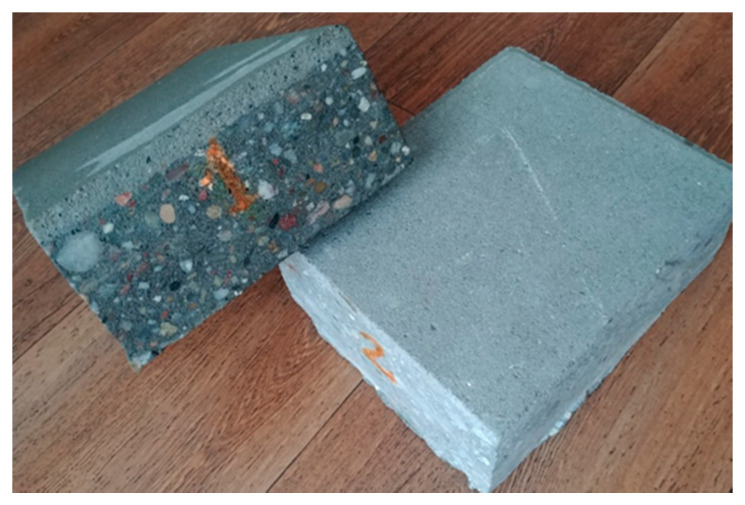
Photocatalytic concrete paving block studied in the research with a novel test method.

In the described research on the novel test method and field campaign, the same samples of photocatalytic concrete paving blocks were investigated. The dimensions of the paving blocks were 500 mm × 500 mm × 70 mm. Photocatalytic concrete was applied only in the 5 mm of the top layer and made from CEM II/A–S 42.5R with nano TiO_2_ (Heidelberg Cement TX Active). The pavement blocks were produced in accordance with standard EN 1339:2003 [27]. In the tests, samples were cut to dimensions of 180 mm × 150 mm × 70 mm to fit into measuring chamber dimensions, as shown in Figure 1.

**Figure 2:**
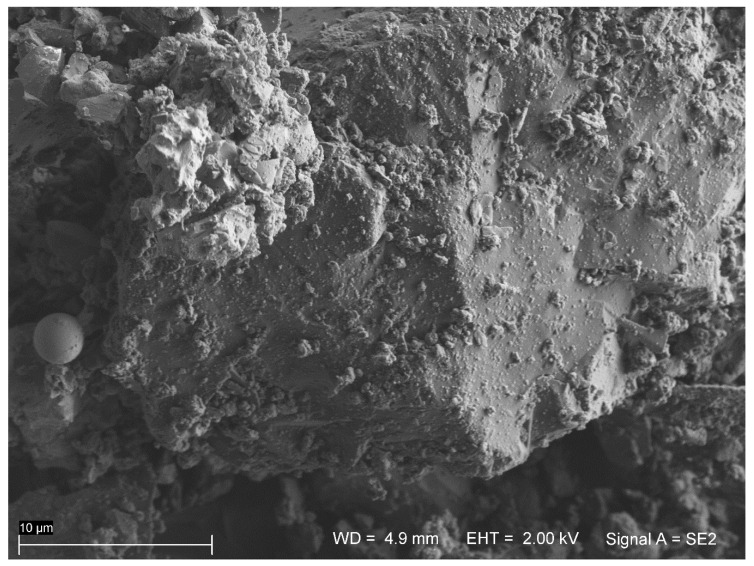
SEM image of the cement sample with TiO_2_ distribution on the cement grain (Everhart–Thornley type detector at ETH = 2.0 kV).

**Figure 3:**
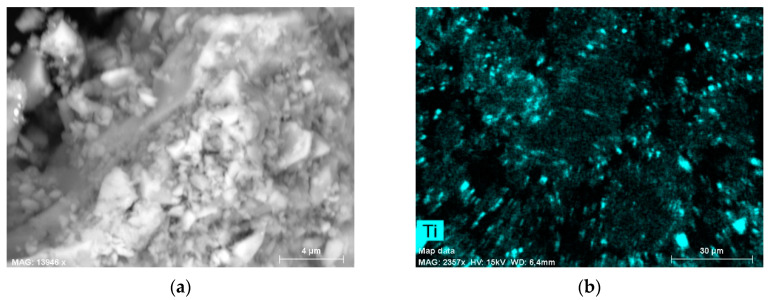
SEM image of the sample surface (**a**) morphology and (**b**) EDS maps of titanium in the investigated region.

In concrete paving blocks, the dominant constituent is an aggregate, and as emphasized by Petrounias et al. [28], aggregate has a major impact on a concrete’s properties. However, the goal of the research was to investigate the concrete pavement’s photocatalytic properties. Accordingly, photocatalytic cement was tested. As the content of the photocatalyst is a key factor affecting the effectiveness of photocatalysis, the distribution of TiO_2_ was verified. According to the manufacturer’s data in CEM II/A–S 42.5R, TX Active cement up to 5% of TiO_2_ P–25^®^ of Degussa was added. To examine the distribution and size of nanoparticles, SEM analysis was applied. A pure sample of cement was investigated. The Sigma VP (Zeiss) equipped with two EDS XFlash 6/10 (Bruker, Oberkochen, Germany) detectors was applied in SEM analysis. To determine the chemical composition, a 120 µm aperture was used, and for high resolution imaging, a 30 µm was used. To ensure the discharge of the electric surface’s charges, the sample was sprayed with carbon and secured with a special strip of electrical charge. The results of SEM analysis (Figure 2 and Figure 3) indicated the grains of nanometric TiO_2_ have sizes of 20–50 nm and form agglomerates on the cement grains. Distribution of TiO_2_ in cement is homogeneous.

The efficiency of photocatalytic paving blocks in air purification was investigated during the field campaign, in situ and with a novel test method. For compatibility of the obtained results, samples were tested according to UNI 11247 [20]. In this reference test, dimension of the sample was in accordance with the standard requirements: 80 mm × 80 mm × 10 mm.

## 3. Generation Park—Field Campaign

**Figure 4:**
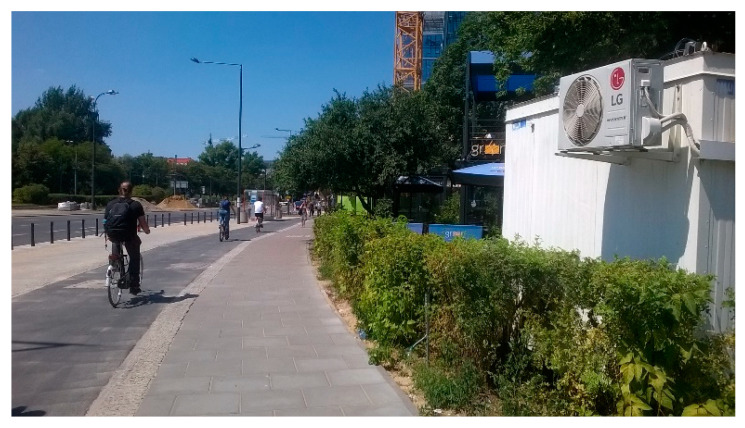
Measuring station at the Daszynskiego roundabout in Warsaw.

In the field campaign (Figure 4), concentrations of NO, NO_2_, and NO*_x_* were measured at the regular sidewalk with typical concrete paving blocks and at the photocatalytic paving blocks, both 400 mm above the pavement surface, where the reaction of photocatalysis occurs. The distance between collecting points was ca. 10 m. In the research, the two API Model 200A NO*_x_* analyzers (Teledyne API, San Diego, CA, USA) were used to determine the NO, NO_2_, and NO*_x_* concentrations. From continuous measurements, 1 min and 1 h means were calculated for further analysis.

**Figure 5:**
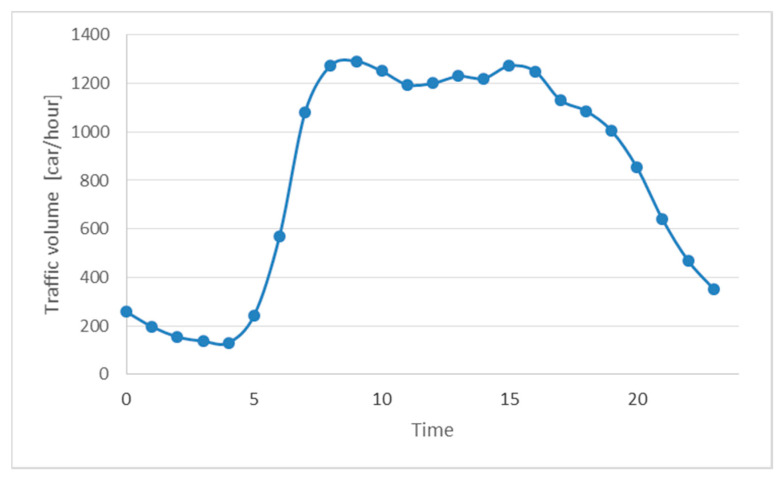
Traffic volume from previous years measured in the vicinity of the Daszynskiego roundabout.

Among several daily tests during the field campaign, a number of days with unfavorable measurement conditions occurred. The major factors affecting the results were wind and traffic intensity, which, during the summer period, were reduced, resulting in relatively low NO*_x_* levels. To achieve representative results, the daily traffic volume from previous years at the Daszynskiego roundabout was analyzed. Data were provided by the Administration of City Roads in Warsaw. An average daily traffic volume (Figure 5) was assumed as a representative level, and only results of the campaign with comparative traffic were assumed in the project.

## 4. Results of Field Campaign

**Figure 6:**
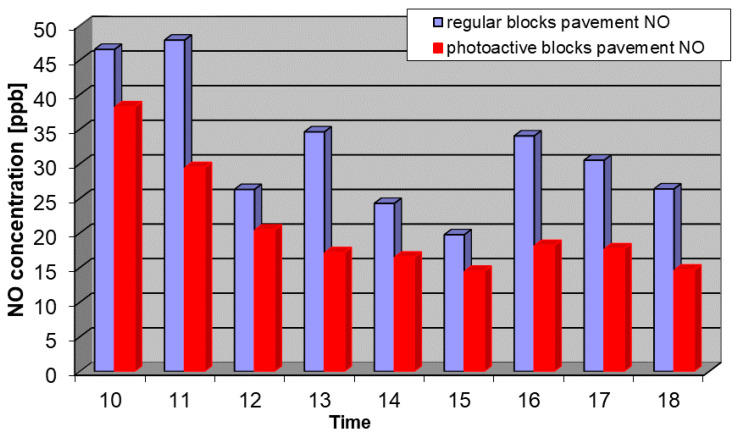
Average concentration of NO on 12 September, 2018 from 10 a.m. to 6 p.m. at the regular pavement (blue) and photocatalytic pavement (red).

**Figure 7:**
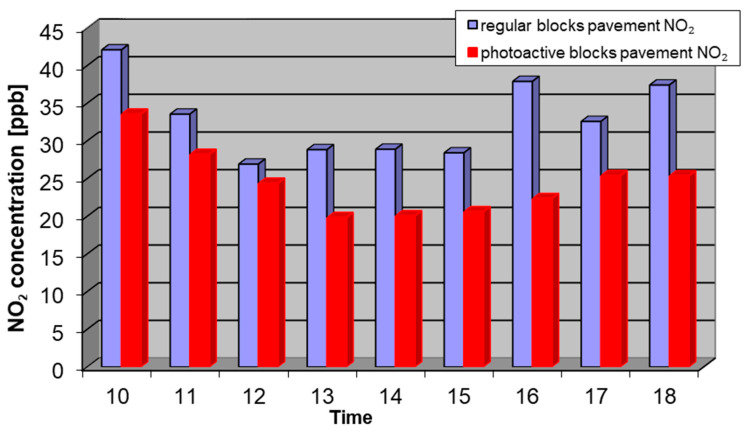
Average concentration of NO_2_ on 12 September, 2018 from 10 a.m. to 6 p.m. at the regular pavement (blue) and photocatalytic pavement (red).

**Figure 8:**
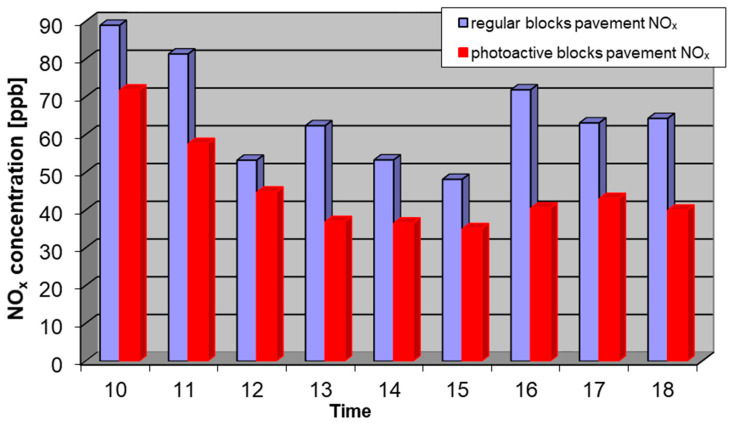
Average concentration of NO*_x_* on 12 September 2018 from 10 a.m. to 6 p.m. at the regular pavement (blue) and photocatalytic pavement (red).

Considering the issues described above, the results of the measurements from 12 September, 2018 with the most representative conditions were chosen. In the tests at the Daszynskiego roundabout, concentrations of NO, NO_2_, and NO*_x_* in the air were measured from two collecting points: above the regular pavement and above the photocatalytic pavement. Measurement results for NO, NO_2_, and NO*_x_* are shown in Figure 6, Figure 7 and Figure 8.

The obtained results indicated that lower values of nitrogen oxide concentration were collected from the photocatalytic pavement than from the normal sidewalk. The difference during the measure period was varied. The average daily values are shown in Table 1. The calculated average daily reduction of nitric oxides was calculated as abatement *A* according to Equation (3):

(3) $$A=\frac{C_{n}-C_{p}}{C_{n}}$$

where A is nitric oxide abatement, $C_{n}$ is the average daily nitric oxide concentration at the normal pavement, and $C_{p}$—average daily nitric oxide concentration at the photocatalytic pavement.

The values of nitric oxides reduction by photocatalytic concrete pavement were higher than the assumed threshold for target large-scale application.

## 5. Removal of Nitric Oxides by Photocatalytic Materials—Novel Test Method

**Figure 9:**
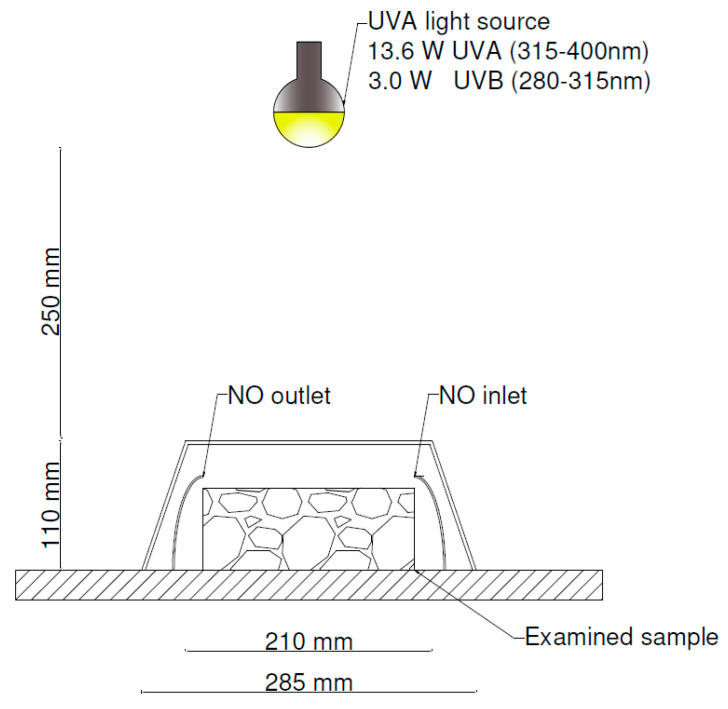
Test setup for laboratory conditions according to Witkowski et al. [29].

According to the findings of the field campaign from the Daszynskiego roundabout and described by Boonen et al. [25], the pollutant gas flow is significantly different from the laminar conditions described in standards such as: ISO 22197–1 [19], UNI 11247 [20], JIS R 1701–1 [21]. Therefore, to provide conditions more supportive of natural gas flow, a test setup described by Witkowski et al. [29] was applied as a base setup in this study. A general scheme of the method is presented in Figure 9.

**Figure 10:**
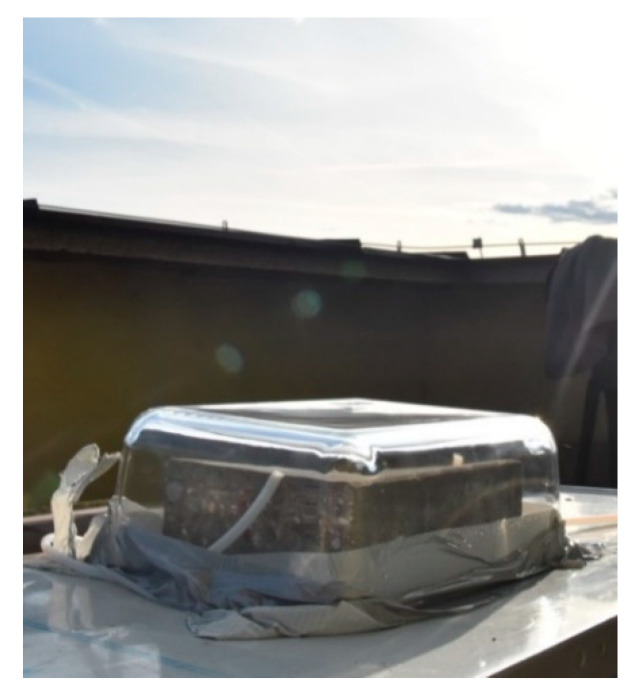
A novel test setup with a natural UV light source.

Although such a test system simulates gas flow reflecting in-situ conditions, artificial light sources provide only selected spectra of UV light with limited UV irradiance. As described by Pierpaoli et al. [14], the results of different light sources characterized by various spectrum peaks between 470–370 nm were different in terms of abatement indexes related to spectra light range. Therefore, to verify the air purification effectiveness of a studied photocatalytic concrete sample, the described setup was placed outdoors and exposed to natural solar light, as shown in Figure 10. During the study, a typical autumn (20 September 2019) UV irradiance level for clear-sky conditions for a latitude of 51.83° N and longitude of 20.78° E occurred for over 4 h of the sample’s exposure to the natural light until sunset. Before the test, an empty glass reactor was filled with a gas up to 100 ppb to verify maximum gas concentration and the tightness of the system. Next, a studied sample was placed in the glass reactor and covered to block the sunlight. The reactor was filled again with gas. As the concentration of the gas reached a maximum, the setup was exposed to the sunlight.

A mixture of synthetic air (consisting of 20% oxygen and 80% nitrogen) and NO gas in a concentration of 100 ppb was used during the study. The gas flow rate was 4 L/min. In the study, NO, NO_2_, and NO*_x_* were measured with the API Model 200A NO*_x_* analyzer. NO*_x_* levels were measured with an accuracy of about ±5%.

The measurement system directly reflects UV-A and visible solar irradiance for natural conditions at a given geographical latitude. However, according to the results of measured spectra of light sources described by Witkowski et al. [29], an applied glass desiccator reduces UVB radiation levels, while UVA radiation, after passing through the glass, is not significantly changed. Furthermore, the system excludes variable wind effects and insufficient pollutant gas concentrations that can occur in environmental conditions. To verify the effectiveness of the considered application, a limited surface of the considered photocatalytic material is needed.

Additionally, photocatalytic concrete’s ability to reduce NO*_x_* levels was measured with a test method described by Witkowski et al. [29] with an artificial light source. Artificial UV light was provided with an OSRAM Vitalux 300W with nominal 13.6 W UV-A (315–400 nm) and 3.0 W UV-B (280–315 nm). Two samples collected from the same concrete pavement element were tested. The time of exposure to artificial UV light was 32–34 min.

As the reference test, the air-purification performance of the sample was measured according to standard UNI 11247 [20] in the Italcementi laboratory in Bergamo.

## 6. Results of the Novel Test Method

**Figure 11:**
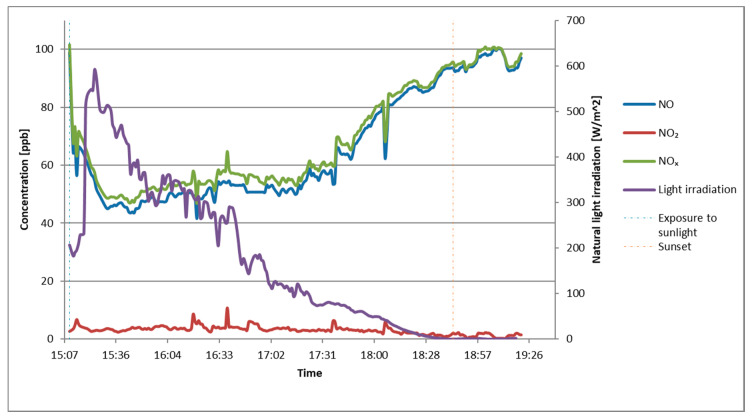
NO, NO_2_, and NO*_x_* abatement with natural UV irradiance measured with the novel test method.

In the study of NO*_x_* abatement with the novel test method, global solar irradiance was measured during the test. The measurement results are shown in Figure 11. Low values of UV irradiance in the first minutes of the test resulted from an occurrence of thick clouds that reduced UV irradiance. Variability of NO*_x_* abatement and natural UV irradiance changes were in good accordance, with a drop in radiance of natural UV light abatement resulting in NO*_x_* decreasing. After sunset, the abatement of NO*_x_* pollutant was nearly zero, and the difference in gas concentration was the result of achieving an equilibrium of gas concentration in the measuring system.

The obtained results of NO_x_ concentration reduction were calculated as an NO*_x_* abatement A according to the Equation (4):

(4) $$A=\frac{C_{B}-C_{L}}{C_{B}}$$

where A is NO_x_ abatement, $C_{L}$ is NO*_x_* reduced concentration in particular time unit, and $C_{B}$ is NO*_x_* concentration measured without UV irradiance (maximum entry value).

Next, the calculated solar zenith angle characteristic to specific dates of the solar position at noon with measured natural UV irradiance and NO*_x_* abatement is presented in Table 2.

**Figure 12:**
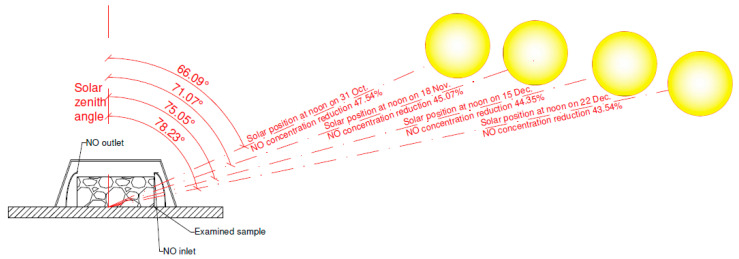
Results of NO*_x_* abatement with the solar zenith angle characteristic to specific dates of the solar position at noon.

The results are schematically presented in Figure 12.

**Figure 13:**
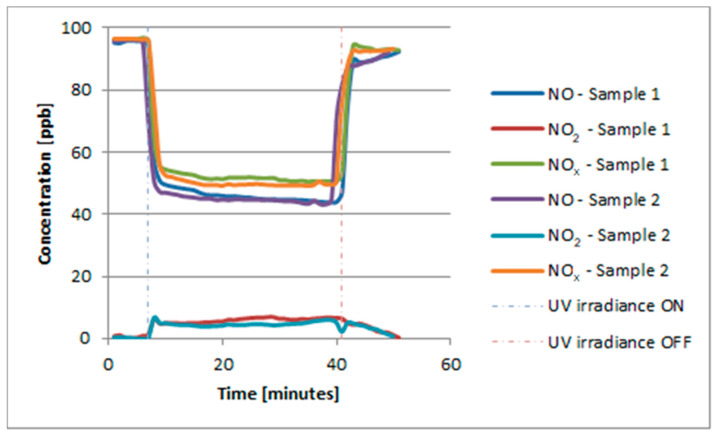
NO, NO_2_, and NO*_x_* abatement with artificial UV irradiance.

In the study with the same setup as described by Witkowski et al. [29] with an artificial light source, the obtained results are presented in Figure 13. The achieved values of NO*_x_* abatement were similar for both samples.

The result of abatement index measures according to standard UNI 11247 [20] is presented in Table 3.

## 7. Conclusions

In this paper, findings from the field campaign in Warsaw were presented. The results confirmed the effectiveness of photocatalytic concrete pavement in the reduction of nitric oxides. The calculated average daily reduction of NO*_x_* was 31%.

Problems that occurred during field measurements motivated the development of a novel test method that was compared with the corresponding test setup with an artificial UV light source and measurement according to standard UNI 11247 [20]. The novel test method uses natural UV light irradiation in a laboratory test setup with a constant gas flow. The shape of the applied desiccators provides nonlinear gas flow that is typical in standardized test setups described in the standards [19,20,21]. The research was conducted on 20 September at the Central Geophysical Observatory of Institute of Geophysics, Polish Academy of Sciences in Belsk (Poland) at a latitude of 51.83° N. In the study, the abatement of the NO_2_, NO, and NO*_x_* concentrations as well as solar UV and global solar light irradiation were measured. According to the described model of geographic location related to solar radiation defined by Folli and Macphee [26], the best results for abatement efficiency are achieved for latitudes below 35°; however, the obtained results with a novel test method indicated that even at low UV light irradiation, the values of measured abatement were above 40%. The calculated solar zenith angle characteristic to specific dates of the solar position at noon with measured natural UV irradiance compared with the result of NO*_x_* abatement demonstrated that, even for the winter solstice, values of abatement were significantly high (43.94%).

The results of NO*_x_* reduction by photocatalytic concrete observed in this study with a novel test method applying natural UV light were lower than the results of an analogous test setup with an artificial UV light source. The gas flow, the size of the specimens collected from the one paving block, and the glass desiccators were the same. The difference was the UV light source. However, the NO*_x_* reduction measured using this novel test method was similar to that obtained in the field campaign.

Although the surface area of the studied samples in the study was significantly larger than the nominal surface area described in UNI 11247 [20] and the achieved results did not correlate, the proposed novel test method provides gas flow corresponding to natural conditions and applies natural light conditions with the entire spectra of UV wavelength range and light irradiation consistent to application conditions. The proposed test setup reduces negative wind effects and insufficient pollutant concentration. The described method combines the advantages of laboratory tests with field tests in specific sunlight conditions.

## Figures and Tables

**Table 1:** Average values of nitric oxide concentrations measured from 10 a.m. to 6 p.m. at regular and photocatalytic pavement at the Daszynskiego roundabout.

Parameter	Regular Pavement			Photocatalytic Pavement		
Nitric oxides	NO	NO_2_	NO*_x_*	NO	NO_2_	NO*_x_*
Average daily concentration (ppb)	32.3	33.0	65.2	20.8	24.4	45.2
Standard deviation	9.7	5.2	13.5	7.9	4.5	12.0
Average daily reduction of nitric oxides (%)	-	-	-	36%	26%	31%

**Table 2:** NO*_x_* abatement measured with a novel method at solar zenith angle characteristic to dates of the solar position at noon for location with a latitude of 51.83° N.

Local Time (hh:min:s)	Solar Zenith Angle (°)	Natural UV Irradiance (290–400 nm) (W/m^2^)	NO*_x_* Abatement (%)	Dates of Solar Position at Noon
15:09:05	60.63	10.89	0 ^1^	15 October
15:25:30	62.55	18.12	43.74	-
15:53:37	66.09	14.12	47.54	31 October
16:10:05	68.29	12.62	44.48	-
16:30:08	71.07	9.4	45.07	18 November
16:57:43	75.05	6.75	44.35	15 December
17:04:32	76.05	5.91	42.92	-
17:19:13	78.23	4.45	43.94	22 December
17:22:11	78.68	4.14	40.66	-
17:47:18	82.46	2.31	32.96	-
18:07:11	85.46	1.18	24.02	-
18:44:37	91.19	0.09	1.85	-

^1^ desiccators covered.

**Table 3:** NO*_x_* abatement index according to Standard UNI 11247 [20].

NO*_x_* Abatement Index
28.2%

## References

[1] EEA (2018). Air Quality in Europe—2018 Report.

[2] Smit R., Kingston P. (2019). Measuring on-road vehicle emissions with multiple instrument including remote sensing. Atmosphere.

[3] Dedele A., Miskinyte A., Cesnakaite I. (2019). Comparison of measured and modeled traffic–related air pollution in urban street canyons. Pol. J. Environ. Stud..

[4] Kwak K., Woo S., Kim K., Lee S., Bae G., Ma Y., Sunwoo Y., Baik J. (2018). On-road air quality associated with traffic composition and street-canyon ventilation: Mobile monitoring and CFD modeling. Atmosphere.

[5] Mulwijk C., Schrijvers P., Wuerz S., Kenjeres S. (2016). Simulations of photochemical smog formation in complex urban areas. Atmos. Environ..

[6] Ganguly R., Broderick B. (2013). Estimation of NOx concentrations for an urban street canyon in Ireland. Int. J. Environ. Eng..

[7] Kang Y., Baik J., Kim J. (2008). Further studies of flow and reactive pollutant dispersion in a street canyon with bottom heating. Atmos. Environ..

[8] Koolen C., Rothenberg G. (2019). Air pollution in Europe. ChemSusChem.

[9] Castellote M., Bengtsson N., Ohama Y., Van Gemert D. (2011). Principles of TiO_2_ photocatalysis. Applications of Titanium Dioxide Photocatalysis to Construction Materials. RILEM State of the Art Reports.

[10] Fujishima A., Honda K. (1972). Electrochemical photolysis of water at a semiconductor electrode. Nature.

[11] Macphee D., Folli A. (2016). Photocatalytic concretes—The interface between photocatalysis and cement chemistry. Cem. Concr. Res..

[12] Fujishima A., Zhang X., Tryk D. (2008). TiO_2_ photocatalysis and related surface phenomena. Surf. Sci. Rep..

[13] Rhee I., Lee J., Kim J., Kim J. (2018). Nitrogen oxides mitigation efficiency of cementitious materials incorporated with TiO_2_. Materials.

[14] Pierpaoli M., Favoni O., Fava G., Ruello M. (2018). A novel method for the combined photocatalytic activity determination and bandgap estimation. Methods Protoc..

[15] Guerrini G., Beeldens A., Crispino M., D’Ambrossion G., Vismara S. Environmental benefits of innovative photocatalytic cementitous road materials. Proceedings of the 10th International Conference on Concrete Pavements.

[16] Hussein A., Al Anbari R., Hassan M. (2018). Humidity Effect on the photocatalytic activity of sustainable cement-based composites. Open Civ. Eng. J..

[17] Beeldens A. An environmental friendly solution for air purification and self-cleaning effect: The application of TiO_2_ as photocatalyst in concrete. Proceedings of the 8th International Conference on Concrete Blocks Paving.

[18] Diamanti M., Lollini F., Pedeferri M., Bertolini L. (2013). Mutual interactions between carbonation and titanium dioxide photoactivity in concrete. Build. Environ..

[19] ISO (2016). ISO 22197-: 2016, Fine Ceramics (Advanced Ceramics, Advanced Technical Ceramics)—Test Method for Air-Purification Performance of Semiconducting Photocatalytic Materials—Part 1: Removal of Nitric Oxide.

[20] UNI (2010). UNI—11247:2010, Determination of the Degradation of Nitrogen Oxides in the Air by Inorganic Photocatalytic Materials: Continuous Flow Test Method.

[21] JSA (2002). JIS TR Z 0018, Photocatalytic Materials–Air Purification Test Procedure.

[22] Guerrini G. (2009). Some observations regarding in-service performance. Photocatalytic paving block surface. Betonw. Fert. Tech..

[23] Guerrini G. (2012). Photocatalytic performance in a city tunnel in Rome: NO_x_ monitoring results. Constr. Build. Mater..

[24] George C., Beeldens A., Barmpas F., Doussin J.F., Maganelli G., Herrmann H., Kleffmass J., Mellouki A. (2016). A impact of photocatalytic remediation of pollutants on urban air quality. Front. Environ. Sci. Eng..

[25] Boonen E., Akylas V., Barmpas F., Boreave A., Bottalico L., Cazauanau M., Chen H., Daele V., de Marco T., Doussin J.F. (2015). Construction of a photocatalytic de-polluting field site in the Leopold II tunnel in Brussels. J. Environ. Manag..

[26] Folli A., Macphee D. Future Challenges for Photocatalytic Concrete Technology. Proceedings of the 34th Cement and Concrete Science Conference.

[27] CEN (2003). EN 1339:2003 Concrete Paving Flags—Requirements and Test Method.

[28] Petrounias P., Giannakopoulou P., Rogkala A., Stamatis P., Lampropoulou P., Tsikouras B., Hatzipanagiotou K. (2018). The effect of petrographic characteristics and physico-mechanical properties of aggregates on the quality of concrete. Minerals.

[29] Witkowski H., Jackiewicz-Rek W., Chilmon K., Jarosławski J., Tryfon-Bojarska A., Gąsiński A. (2019). Air purification performance of photocatalytic concrete paving blocks after seven years of service. Appl. Sci..

